# Society Proceedings

**Published:** 1902-01

**Authors:** 


### SOCIETY PROCEEDINGS.

### KEYSTONE VETERINARY MEDICAL ASSOCIATION.

THE December meeting of this Association was held at the northwest corner of Broad and Filbert Streets, December 10, 1901, with the following members of the profession in attendance: Drs. Houldsworth, Pearson, Eves, Goentner, Hoskins, Marshall, Rhoads, Underhill, Ridge, Williams, Powell, Lintz, Sellers, Harger, Carter, Gillilan, Seidel, of the veterinary profession, and Dr. M. P. Ravenel, of the medical fraternity. Thirty-five students of the Veterinary Department of the University of Pennsylvania were also in attendance. After the regular business was transacted Dr. Pearson delivered an address on "Observations from Foreign Veterinary Colleges," giving all the details of the buildings, business management, students' homes, as well as the connections with State or government.

He took as a basis the Berlin school, which is the oldest in Germany, being founded 110 years ago. It stands on the ground on which it was founded, a tract of land of twenty-five acres, on the outskirts of the town; but since its foundation the city has grown so that residences and stores are built all around the college. They are at present building a new structure, to be used exclusively for teaching anatomy. On the first floor are rooms for janitor, storage, and freezing. On the second floor are rooms for dissecting; these rooms are supplied with large skylights and stationary tables. The third floor is used for a museum of anatomy.

Another new building is that used for the department of hygiene, under Professor Ostertag, who teaches meat and milk-inspection and bacteriology. Every appliance is to be found here, and this department is directly under the professor and six assistants. Connected with this building are stable accommodations for experimental animals for antitoxin, and for diseases not due to bacteria—helminths, etc.—also a large dog hospital.

There are rooms for operating purposes, for clinics, and also for a pharmacy. A novel feature is a large cow stable, built on model plans, for forty cows of the most improved types and breeds. This is kept and managed as a model dairy, and the milk is sold in the neighborhood. The clinics and hospitals for large animals are all one-story buildings, arranged in a court, consisting of blacksmith shop, operating-room, clinic rooms, special rooms for surgical cases, riding school, exercise rooms, isolation wards; there is also provided a ward for animals suffering from internal and infectious diseases. The courtyard is covered with soft sand, through which horses are led to test for soundness.

The capacity of the hospital is about one hundred horses. From twenty to fifty animals are treated in the out-clinic daily.

A large dormitory building for military students is provided and supported by the government. These students must serve as long in the army as they spend in the college.

The other schools are on general plan very much as Berlin; some teach special branches, to the detriment of others. The Hanover School is the newest equipped. The Dresden School is hampered for room. The Munich

School has been recently re-equipped. At Stuttgart is to be found the well-known Hoffman table for operations on horses, on which 1200 horses have been operated upon in two years.

The Budapest (Hungary) School is comparatively new, having been built about fifteen years ago. This school is supported by the government. It must be admitted that schools supported by governments are better equipped than those which are not, which accounts in a large measure for our inferior schools here in the United States.

The London School, founded by Bell in 1792, stands on leased ground, and is supported by friends of the school and students. The examinations are conducted by a committee appointed by the Royal Veterinary Society, and graduates who pass this examination are admitted to this Society. Before concluding this valuable discourse Dr. Pearson drew some valuable and interesting comparisons with our schools, and pointed out the advantages and disadvantages found here, but thought they were gradually developing toward Continental systems.

Dr. M. P. Ravenel, bacteriologist for the Pennsylvania Live-stock Sanitary Board, having been abroad as a representative from that Board at the "Tuberculosis Congress at London," was the next speaker for the evening. Dr. Ravenel was one of the first men to take issue with Professor Koch's statement as to the communicability of tuberculosis from cattle to man. He says, in part, that Koch's statement was entirely too sweeping and too radical; he overlooked the statistics of other pathologists; he was irrational and unreasonable, for the following reasons: 1. That no animal is absolutely immune to tuberculosis. 2. Several cases definitely known, and many cases not clearly proven, have been placed on record in which the infection was traced to the bovine species. 3. It has been definitely proved that any infectious or contagious disease which attacks this species of animals is also transmissible to man. Dr. Ravenel then explained that all the findings and experimental work brought forward by the "Tuberculosis Congress" were nothing new, for under the plans which Dr. Leonard Pearson formulated for experimental investigation for the S. L. S. B. of Pennsylvania on tuberculosis covered all the essential features, and in fact some of them were entirely new to foreign experts. Dr. Ravenel thinks that this agitation will bring good results, for although there is now a storm of protest, it will also result in vast experimentation all over the world, and thus end in some definite scientific conclusion.

A vote of thanks was tendered Drs. Pearson and Ravenel by the Association for their well-prepared and instructive addresses. The next in order was a carefully prepared paper on "A Case of Tetanus, with Antitoxin Treatment," by Dr. B. N. Underhill, of Media. The doctor used antitoxin in large doses, which seemed to ameliorate the condition, and the horse is evidently making a prompt recovery. This paper caused a great amount of discussion, which was entered into by Drs. Williams, Harger, Sellers, Ranck, Eves, Ridge, Goentner, Pearson, Marshall, Hoskins, Rhoads, and others.

The consensus of opinion favored tetanus antitoxin as a valuable prophylactic measure, but not always a reliable curative measure. Dr. Pearson then made a motion to have the President appoint a Committee to frame resolutions to be sent to Governor Stone, favoring Dr. Hoskins' reappointment on the State Examining Board. Carried. The Committee appointed consisted of Drs. Goentner, Marshall, and Ranck. The following committees were appointed by President Rhoads:

Committee on Milk: W. H. Hoskins, Chairman, H. P. Eves, W. L. Rhoads, W. H. Ridge, Leonard Pearson.

Committee on Diseases: C. J. Marshall, Chairman, E. M. Ranck, J. R. Mahaffey, J. D. Houldsworth, J. M. Carter.

Programme Committee: E. M. Ranck, Chairman, James Mecray, B. M. Underhill, W. S. Kooker, A. F. Schreiber.

Press Committee: J. M. Carter, Chairman, C. T. Goentner, Charles Lintz, Francis Bridge, Thomas B. Rayner.

After the announcement of the above committees the Programme Committee secured papers for the January meeting from Drs. C. J. Marshall and S. J. J. Harger, the subjects of which will be furnished later. A motion to adjourn was carried to meet the second Tuesday evening in January.

E. M. RANCK,
Secretary.

### CALL FOR THE FOURTEENTH ANNUAL MEETING OF THE IOWA STATE VETERINARY MEDICAL ASSOCIATION.

THE next annual meeting of this Association will be held in the club-room, Savery Hotel, Des Moines, Iowa, on Tuesday and Wednesday, February 11 and 12, 1902.

The members of the Association are earnestly requested to attend and enter into the work and deliberations of the meeting. The meeting can be made a success only through the co-operation of the members of the Association. Their individual success and that of the profession depend in a large measure upon the strength of the Association. It is their duty to make a strenuous effort to further its interests whenever an opportunity presents itself. This is one of those opportunities.

Attention is directed to the fact that the first session is announced for 9.30 A. M. on Tuesday, February 11th, and must begin then in order that the programme may be completed within the time limit; also, the papers will not be presented until the afternoon of Wednesday, February 12th, at which time the election of officers and appointment of committees will take place. Thus it can be seen that the members will need to leave home at such a time as will enable them to reach the place of meeting by the time of the opening of the first session, and to so arrange their affairs that they need not leave the meeting until the close of the last session.

Those who regularly attend these meetings unanimously testify to the benefits they receive in the way of knowledge gained from the reports, papers, and discussions, and to the stimulation and inspiration afforded by personal contact and acquaintance with others who are devoting themselves to the same life work. The greater the attendance the greater these individual benefits will be. Everyone who attends will be amply repaid for the time and money expended in so doing.

All graduated veterinarians who are not members are cordially invited to be present at the meeting and to join in active membership in the Association. All others who are interested in the meeting are given a cordial invitation to be present at its sessions.

*Programme—First Day.* "Reports of Cases—Anthrax in the Horse," Dr. W. Hamilton; "Chronic Atrophic Orchitis in the Bull," Dr. H. C. Simpson; "Ravages of the Strongylus Tetracanthus," Dr. S. H. Kingery; "Urethral Calculus and Paralysis of the Penis," Dr. E. G. Marten; "A Cow Case," Dr. G. P. Statter; "Typhoid Fever in a Horse," Dr. L. U. Shipley; "A Case in Practice," Dr. A. H. Quinn; "Impaction of the Small Colon," Dr. J. Vincent; "Report of a Case," Dr. J. Thomsen.

*Programme—Second Day.* In the forenoon a clinic will be conducted at the infirmary of Dr. H. E. Talbot, No. 607 Des Moines Street. The demonstrations have been distributed among a number of good operators, and promise to be very instructive. Papers: "Abortion in Cows," Dr. P. Malcolm; "Amputation of a Bull's Penis," Dr. G. M. Walrod; "Parturition Cases," Dr. W. Drinkwater; "Obstinate Constipation," Dr. J. W. Griffith; "External Ulcerative Anovulvitis," Dr. S .T. Miller; "Rabies," Dr. D. E. Baughman; "Symptoms of Rabies," Dr. S. H. Johnston; "The Trials of the Veterinary Board," Dr. H. E. Talbot; "Cæsarean Section," Dr. H. L. Stewart; Volunteer Papers; Election of Officers; Report of Committee on Resolutions; Appointment of Committees; Adjournment.

JOHN J. REPP,
Secretary pro tem.

### THE ONTARIO VETERINARY ASSOCIATION.

THE annual meeting of this Association was held in the Ontario Veterinary College, Toronto, Canada, on Friday, December 20, 1901.

In the absence of the President, Dr. H. S. Wende, the First Vice-President, Dr. J. H. Tennant, took the Chair, opened the meeting at 11 A. M., and called for the usual routine business, viz., the reading of the minutes of the last meeting, and the Secretary's, Treasurer's, Registrar's, and Auditor's reports, which were read and approved.

The following new members were then duly proposed and accepted: Messrs. W. J. R. Fowler, V.S.; C. Brind, V.S.; J. H. Engel, V.S.; A. D. Stewart, V.S., and R. A. Milne, V.S.

A number of communications were read.

It was moved by Dr. O'Neil, and seconded by Prof. Reed, of the Guelph Agricultural College, that the Secretary be instructed to write to Mrs. R. S. Huidekoper, and express to her and her family the deep sympathies of the members of this Association with them in the sad loss they have recently sustained by the death of the late eminent veterinary surgeon, R. S. Huidekoper.

The meeting then adjourned to meet after luncheon

The meeting re-opened after luncheon, the President, Dr. H. S. Wende, in the Chair. The President on opening the meeting explained his absence as being in consequence of the non-arrival of his train on time, and after a short address called for the reading of papers.

Prof. J. H. Reid, of Guelph, described a case of urethral calculus in a gelding. He removed it by a surgical operation. The operation was followed by considerable swelling of the penis, which soon subsided. A short time afterward another calculus appeared in the urethra; this one was much larger than the first. He removed this also by a similar operation. Both the calculi were situated near the ischial arch. There was some constriction of the urethra following the operations, but complete recovery resulted.

Dr. Orr Graham, V.S., read a paper on "Œdema of the Tongue of the Horse," of which he had seen some cases. The conditions existing were rapid swelling of the tongue, so great that it completely filled the cavity of the mouth, pressing the jaws widely apart, and the tongue protruding some distance, accompanied by an abundant discharge of fluid from the mouth and nostrils. In two instances the animals died from suffocation. The disease came on very rapidly and without any cause that could be discovered. When the swellings began to recede the recoveries were rapid and permanent.

Dr. S. E. Boulter read an excellent paper on the necessity for observing kindness and gentleness in the treatment of our patients, and although severe and painful operations may sometimes be necessary, everything possible should be done to alleviate pain and obviate suffering.

Dr. D. K. Smith, Professor of Pathology and Microscopy in the Ontario Veterinary College, read a very useful and practical paper on preserving and preparing pathological specimens. He gave demonstrations of the use of the microtome, also of staining and mounting microscopical specimens.

Dr. W. J. Wilson read a paper on the treatment of tetanus. He claimed that "serum therapy" has not brought the good results that its advocates anticipated.

The reading of the papers was followed by interesting discussions upon them, in which many members participated; and it was moved that the thanks of the meeting be tendered to those gentlemen who had contributed the papers.

The sum of $25 was appropriated for a medal to be competed for by the students of the Ontario Veterinary College at the approaching spring examination.

The Secretary was instructed to send postal cards to the duly qualified veterinary practitioners in Ontario who have not registered in accordance with the act of incorporation of the Ontario Veterinary Association, requesting them to register; also to get a number of new copies of the Ontario Veterinary Register printed.

The following gentlemen were appointed to read papers at the next annual meeting: Messrs. L. A. Wilson, J. D. O'Neil, W. J. Wilson, S. E. Boulter, R. A. Milne, C. Brind, Dr. D. K. Smith, and J. H. Tennant.

The following is the list of officers for the ensuing year: J. H. Tennant, President; W. Steele, First Vice-President; W. Lawson, Second Vice-President; C. H. Sweetapple, Secretary and Treasurer.

Directors: Messrs. C. Brind, J. H. Engel, S. E. Boulter, L. A. Wilson, J. H. George, F. G. Hutton, R. A. Milne, and F. Daly.

Auditors: C. Elliott and J. H. Reed.

Delegates to the Industrial Fair, Toronto: Prof. A. Smith and C. Elliott.

Delegates to the Western Fair, London: Messrs. J. H. Tennant and J. D. O'Neil.

C. H. Sweetapple, V.S.,
Secretary.

